# Investigation of the Association Between 584C/T Polymorphism of EL Gene and Risk of Premature Coronary Artery Disease in Fars Province

**DOI:** 10.15171/jcvtr.2015.25

**Published:** 2015

**Authors:** Samane Toosi, Sara Senemar, Zeinab Ahmadi, Salma Radmanesh

**Affiliations:** ^1^ Human Genetic Research Group, Iranian Academic Center for Education, Culture & Research (ACECR), Fars Branch, Shiraz, Iran; ^2^ Department of Cardiology, Shiraz University of Medical Sciences, Shiraz, Iran

**Keywords:** Coronary Artery, Endothelial Lipase, Polymorphism, 584C/T

## Abstract

*Introduction:* Endothelial lipase (EL) is a protein from the triglyceride lipase family which plays an important role in high-density lipoprotein (HDL) metabolism. One of the most frequently studied variants is 584C/T which causes the amino acid threonine at codon 111 to convert to isoleucine. Many studies have shown the association of this variant with HDL-C level and CAD disease.

*Methods:* The population of this study consists of 140 patients (all males) with angiographically confirmed coronary artery disease (CAD) and 80 controls. Polymerase chain reaction and restriction fragment length polymorphism (PCR-RFLP) was carried out for genotyping of LIPG 584C/T. Data were analyzed using SPSS.

*Results:* The results of the study indicated that the frequency of T allele was significantly lower among CAD patients than among controls (0.27 vs 0.36, *P* = .004). However, no significant correlation was found between the 584C/T variant and serum HDL-C level. Multivariate regression analysis confirmed that the T allele is significantly associated with CAD disregarding the age, hypertension, hypercholesterolemia, diabetes and HDL-C (OR = 0.494, 95% CI = 0.253- 0.968, *P* =.040).

*Conclusion:* It was concluded that the T allele was associated with protection from CAD in Fars province independent of HDL-C level.

## Introduction

One of the most important risk factors for coronary artery disease (CAD) is dyslipidemia which is comprised of high levels of plasma total cholesterol (TC), triglyceride (TG), low-density lipoprotein (LDL) and low levels of high-density lipoprotein (HDL). HDL-C levels are inversely correlated with CAD.^1-3^ High HDL-C levels are associated with the protection from CAD and low HDL-C levels are related with elevated risk of CAD.^4^ Endothelial lipase (EL) is a protein from the triglyceride lipase family which is expressed in the endothelial cells and tissues like the liver, lung, macrophage, testis, ovary and placenta. The other members of triglyceride lipase family are hepatic lipase and lipoprotein lipase which have a high molecular homology to EL.^1,5,6^ EL acts as a heparin binding protein that is connected to the luminal endothelial surface via heparin sulfate proteoglycans. EL has low triglyceride lipase and high phospholipase activities.^4^ EL is a regulator of plasma HDL-C levels and can modulate plasma HDL-C levels inversely.^1,7^ Many studies have revealed that inhibition of EL by antibodies or by gene deletion of LIPG has caused increased HDL-C levels in human and mouse. Therefore, as expected, a decrease in EL expression and activity leads to increases in HDL-C levels and possibly a decrease in CAD risk.^1,8,9^ Jaye et al^10^ demonstrated that overexpression of EL in the liver by adenovirus-mediated gene transfer is negatively associated with HDL-C levels and leads to a decrease in the HDL-C level. Moreover, in the study by Shimizu et al^11^ on the male LIPG knockout mouse the level of HDL-C showed a 57% increase as compared with wild-type controls. LIPG gene encoded a protein of 500 residues, located on chromosome 18 and containing 10 exons and 9 introns. Several genetic variants have been recognized in the LIPG gene.^4^ Among these, 584C/T is the most frequently studied variant that can alter the amino acid threonine, converting into isoleucine at codon 111.^12,13^ Some studies supported the idea that the 584C/T variant was inversely correlated with serum HDL-C levels^14,15^ but in other studies this association was not found.^11,16^ So far, the correlation between 584C/T variant and the risk of CAD and HDL-C level has not been established in Fars province. Therefore, in the present study the effect of 584C/T variant on the risk of CAD and HDL-C levels was investigated in Fars province.

## Material and Methods

### Study Population

A total of 220 subjects consisting of 140 patients (all males) and 80 controls aged <80 years were recruited from Namazi hospital of Shiraz University of Medical Sciences. The prevalence of CAD is different between males and females. The sample size of women was very small and they were excluded from the study. The subjects had undergone coronary angiography and left ventriculography and were divided into 2 CAD patients and control groups. The subjects who had ≥50% stenosis in at least one coronary artery were defined as CAD patients and the subjects with <30% stenosis in all their major vessels were defined as controls.

All subjects were monitored as to conventional risk factors for CAD, including diabetes mellitus, hypertension and smoking. Hypertension was defined as blood pressure of 140/90 mm Hg or use of blood pressure lowering medication. Diabetes was defined as the use of insulin or fasting glucose level ≥125 mg/dl. The subjects who smoked >1 cigarette per day were defined as smoker. The hypercholesterolemia was marked by fasting cholesterol level >200 mg/dl or medicine treatment. Approximately, 5 ml fasting blood samples were obtained to determine the levels of TC, TG, LDL-C and HDL-C by enzymatic methods with commercially available kits.‏

## Genotyping

Genomic DNA was isolated from the peripheral blood leukocyte through phenol-chloroform methods. Polymerase chain reaction and restriction fragment length polymorphism (PCR-RFLP) was carried out for genotyping of LIPG 584C/T. The polymorphic site on exon 3 was amplified by PCR, using the following primer pair:

F: 5′-ACAAACCCAAAGCTACACAGACT-3′

R: 5′- ACCACAACTACATTGGCGTC TTTCTCTCAT-3′

A single base A was inserted at position 35 in the reverse primer to create a restriction site for restriction endonuclease NdeI. The NdeI restriction enzyme was used to digest the 254-bp product and then identified by electrophoresis on 1.5% agarose gel followed by ethidium-bromide staining. In subjects with T allele, NdeI digested the 254-bp product and produced 217 and 37-bp segments while in those with C allele there was no site for NdeI.^4^

### Statistical Analysis

SPSS version 15 was used for statistical analysis. Continuous variables were expressed as mean±SEM, and qualitative variables were presented as percentages. Student *t* test was carried out for calculating the differences of continuous variables between cases and controls. Categorical variables were presented using frequency counts and compared by chi-square test. Univariate and multiple logistic regression analysis were used to predict the probability occurrence of CAD. We calculated Odds Ratio (OR) and 95% CI. Statistical significance was expressed as *P* <.05.

## Results

The characteristics of the case and control groups are expressed in Table 1. No significant difference was found between the case and control groups in age (*P* >.05), indicating that the subjects were well matched. The prevalence of hypertension, hypercholesterolemia, smoking, diabetes was higher in CAD patients than control subjects. The CAD patients had also higher levels of TC, TG, LDL but lower HDL, as compared with control subjects. There was no significant difference in hypercholesterolemia, diabetes, and TC between the two groups. 584C/T genotypes distribution and allele frequencies in 2 groups of CAD patients and control subjects are shown in Table 2. The number of subjects with TT genotype was small; thus, the CT and TT genotype groups were considered as T allele carriers for statistical analysis. The T allele frequency was lower in CAD patients than in control subjects. The frequencies of the T and C allele were 0.36 and 0.64, respectively in the control group while they were 0.27 and 0.73 in the case group. The TT+CT genotype was significantly higher in the control group than in CAD patients. The HDL level according to 584C/T genotypes in CAD patients and control subjects, were shown in Table 3. There was no significant differences between the 584C/T genotype and HDL-C level. Univariate and multiple logistic regression analyses were used to investigate the relationship between the risk factors and CAD. The results are shown in Table 4. In univariate analysis, the risk for CAD in T allele carriers was significantly reduced. (OR = 0.440). Multiple logistic regression model 1 including 584C/T genotype, age, hypertension, hypercholesterolemia and smoking, was conducted and the OR for subjects with the T allele was 0.464. Multiple logistic regression model 2 including 584C/T and HDL-C level indicated that the T allele had a protective effect on CAD disease but it was reduced. (*P* =.011). Multiple logistic regression model 3 included all the 5 variables, and the *P* value remained significant (*P* = .040).

**Table 1:** Baseline Characteristics of Cases and Controls

**Risk Factors**	**CAD** **n = 140**	**Controls** **n = 80**	*** P *** ** Value**
AGE (y)	46 ± 4.2	45 ± 3.8	.200
Hypertension, n (%)	62 (44.3%)	19 (23.8%)	.002
Hypercholestremia, n (%)	44 (31.4%)	21 (26.2%)	.250
Smoking, n (%)	82 (58.6%)	29 (36.2%)	.001
Diabetes, n (%)	22 (15.7%)	14 (17.5%)	.430
TG (mg/dl)	212.1 ± 111.2	174.5 ± 109.7	.016
TC (mg/dl)	157 ± 53.6	144.2 ± 63.4	.091
LDL (mg/dl)	150.1 ± 50	122.9 ± 73.1	.001
HDL (mg/dl)	43.9 ± 16.6	53.3 ± 21.4	<.001

Data are as mean ± SEM or number (%) of patients. Abbreviations: CAD, coronary artery disease; TC, total cholesterol; TG, triglyceride; HDL-C, high-density lipoprotein-cholesterol; LDL-C, low-density lipoprotein-cholesterol.

**Table 2:** Genotype and Allele Distribution of the 584C/T Variant in Cases and controls

	**CAD** **n = 140**		**Controls** **n = 80**		*** P *** ** Value**
CT	70	52.1%	46	71.2%	.004
TT	3		6		
CC	67	47.9%	28	28.8%	
T allele Frequency	76	27.1%	52	32.5%	.004
C allele Frequency	204	72.9%	108	67.5%	

Data are number (%) of patients. Abbreviations: CAD, coronary artery disease;

**Table 3:** HDL-C Levels With Respect to the 584C/T Genotype

	**HDL-C mg/dl**	*** P *** **Value**
CAD		
TT+CT	42.8 ± 11.59	.060
CC	18.5 ± 46.14	
Control		
TT+CT	19.2 ‏± 62.94	.360
CC	37.2 ‏± 57.43	

Data are mean ± SEM (mg/dl). Abbreviations: CAD, coronary artery disease; HDL-C, high-density lipoprotein-cholesterol.

**Table 4:** Univariate Analysis and Multiple Logistic Regression Analysis for CAD Risk

	**OR**	**95% CI**	*** P *** **Value**
T allele carriers (TT+CT)	0.440	0.791 - 0.244	.006
T allele carriers (TT+CT)	0.464	0.858 - 0.251	.014
Hypertension	2.478	4.690 - 1.309	.005
Smoking	2.596	4.686 - 1.439	.002
T allele carriers (TT+CT)	0.453	0.83 - 0.264	.001
HDL-C	0.978	0.99 - 0.980	<.001
T allele carriers‏ (TT+CT)	0.494	0.253 - 0.968	.040
HDL-C	0.993	0.987 - 1.000	.045
Hypertension	2.694	5.511 - 1.317	.007
Smoking	2.448	4.690 - 1.278	.007

Abbreviations: CAD, coronary artery disease; HDL-C, high-density lipoprotein-cholesterol; OR, odds ratio.

## Discussion

In this study, we investigated the effect of 584C/T variant on the risk of CAD in Fars province. The results indicated that the 584C/T genotypes in LIPG gene are significantly associated with CAD in our population‏. The frequency of T allele in CAD patients was (0.27) and in the control subjects it was (0.36), indicating that the T allele variant is considered as a CAD reducing factor and is statistically associated with CAD, although there was no correlation between the T allele and HDL-C level. This result is consistent with that of the study by Shimizu et al on Japanese people.^11^ However, our observation is in conflict with the results of the study by Na-Ping Tang which demonstrated the T allele carriers had significantly elevated plasma HDL-C levels.^4^ delemos et al^12^ performed the study including three groups of 176 black controls, 165 white controls and 123 white subjects with high HDL-C. They demonstrated that the T allele was not significantly associated with plasma HDL-C levels. In another study by Yamakawa-Kobayashi et al^16^ has shown that there was no correlation between serum HDL-C levels and the T allele in Japanese school-aged children. EL is expressed in endothelial cells surface; and through interaction with heparin sulfate proteoglycans it can promote the adhesion of the monocyte to the vascular endothelium.^17^ Studies on the mouse and human have shown that EL is a major regulator of HDL metabolism.^4,18^ It has been demonstrated in transgenic mouse that EL inversely correlates with HDL-C levels and high expression of EL leads to reduction of the HDL-C levels.^19^ In the study by Jaye et al^10^ it was shown that over-expression of EL is a cause of reduced plasma HDL-C levels in the mice. Choi et al^20^ found that loss of function in EL variants leads to an increases in HDL-C levels. In this study, we performed univariate logistic regression analysis and according to the results protective effect of the T allele on CAD disease was confirmed (OR = 0.44). So, the subjects with CT and TT genotype had a reduced risk of CAD compared with CC genotype. In addition, the effects of other factors on occurrence of CAD were analyzed by 3 models of multiple logistic regression. Multiple logistic regression model 1 including 584C/T genotype, age, smoking, hypertension, diabetes and hypercholesterolemia revealed that the T allele protectively effect CAD and the relationship between T allele and CAD was significant. (OR= 464, 95% CI: 0.858-0.251, *P* =.0140). Multiple logistic regression model 2 including 584C/T genotype and HDL-C showed that the value of OR did not change significantly; therefore, the HDL-C levels did not influence the susceptibility to the CAD and the protective effect of T allele remained significant (OR = 0.453, 95% CI: 0.837-0.264, *P* =.001). the results of multiple logistic regression model 3 including all variables of age, hypertension, smoking, diabetes, hypercholesterolemia and HDL-C indicated that the T allele had a protective effect on CAD disease but it was reduced. The data of this study confirmed that the T allele was significantly associated with CAD disregarding age, hypertension, hypercholesterolemia, diabetes and HDL-C (OR = 0.494, 95% CI: 0.253-0.968, *P* =.040).

## Conclusion

In conclusion, it was confirmed that there was an association between the T allele and protection from CAD in Fars province.

## Acknowledgements

The authors acknowledge financial support from human genetics research group of ACECR, Fars branch and the hospitals staff for helping us. The authors would like to thank Dr. Nasrin Shokrpour at Center for Development of Clinical Research of Nemazee hospital for editorial assistance.

## Ethical issues

The ethics committee of Iranian Academic Center for Education, Culture and Research (ACECR) approved the protocol of this study, and it was conducted based on Helsinki Research Ethics and good clinical practices. All patients participating in this study provided written informed consent and all data were collected by a single nurse.

## Competing interests

Authors declare no conflict of interest in this study.

## References

[1] Liu WY, Yin RX, Zhang L, Cao XL, Miao L, Wu DF, Aung LH (2010). Association of the LIPG 584C>T polymorphism And serum lipid levels in the Guangxi Bai Ku Yao And Han populations. J Lipids Health Dis.

[2] Jeppesen J, Hein HO, Suadicani P, Gyntelberg F (1998). Triglyceride concentration And ischemic heart disease: aneight-year follow-upinthe Copenhagen MaleStudy. J Circ.

[3] Satoh H, Nishino T, Tomita K, Tsutsui H (2006). Fasting triglyceride is a significant Risk factor for coronary artery disease in middle-aged Japanese men: results from a 10-years cohort study. J Circ.

[4] Tang NP, Wang LS, Yang L, Zhou B, Gu HJ, Sun QM (2008). Protective effect of an endothelial lipase gene variant on coronary artery disease in a Chinese population. J Lipid Res.

[5] Miljiccovic-Gacic I (2004). Association of hepatic lipase and endothelial lipase polymorphism with variation NMR lipoprotein subclasses in Caucasian, African-American and African-Caribbean older men. University of Pittsburg.

[6] Hirata K, Dichek HL, Cioffi JA, Choi SY, Leeper NJ, Quintana L (1999). Cloning of a unique lipase from endothelial cells extends the lipase gene family. J Biol Chem.

[7] Yasuda T, Ishida T, Rader DJ (2010). Update on the role of endothelial lipase in high-density lipoprotein metabolism, reverse cholesterol transport, and atherosclerosis. J Circ.

[8] Ishida T, Choi S, Kundu RK (2003). Endothelial lipase is a major determinan of HDL level. J Clin Invest.

[9] Ma K, Cilingiroglu M, Otvos JD, Ballantyne CM, Marian AJ, Chan L (2003). Endothelial lipase is a major genetic determinant for high-density Lipoprotein concentration, structure, and metabolism. J PNAS.

[10] Jaye M, Lynch KJ, Krawiec J, Marchadier D, Maugeais C, Doan K (1999). A novel endothelial-derived lipase that Modulates HDL metabolism. J Nat Genet.

[11] Shimizu M, Kanazawa K, Hirata K (2007). Endothelial lipase gene polymorphism is associated with acute myocardial infarction, independently of high-density lipoprotein-cholesterol levels. J Circ.

[12] deLemos AS, Wolfe ML, Long CJ, Sivapackianathan R, Rader DJ (2002). Identification of genetic variants in endothelial Lipase in persons with Elevated high-density lipoprotein cholesterol. J Circ.

[13] Paradis ME, Couture P, Bossé Y (2003). The T111 I mutation in the EL gene modulates the impact of dietary fatonthe HDL profile in women. J Lipid Res.

[14] Halverstadt A, Phares DA, Ferrell RE, Wilund KR, Goldberg AP, Hagberg JM (2003). High-density lipoprotein-cholesterol, its subfractions, and responses to Exercise training are dependent on endothelial lipase genotype. J Metabolism.

[15] Mank-Seymour AR, Durham KL, Thompson JF, Seymour AB, Milos PM (2004). Association between single-nucleotide polymorphisms in the Endothelial lipase (LIPG) gene and high-density lipoprotein cholesterol levels. J Biochim Biophys Acta.

[16] Yamakawa-Kobayashi K, Yanagi H, Endo K, Arinami T, Hamaguchi H (2003). Relationship between serum HDL-C levels and common genetic variants of the endothelial lipase gene in Japanese school-aged children. J Hum Genet.

[17] Kojma Y, Hirata K, Ishida T (2004). Endothelial lipase modulates monocyte adhesion to the vessel wall: A potential role in inflammation. J Biol Chem.

[18] Maugeais C, Tietge U J, Broedl UC (2003). Dose-dependent acceleration of high density lipoprotein catabolism by endothelial lipase. J Circ.

[19] Broedl UC, Maugeais C, Marchadier D, Glick J M, Rader DJ (2003). Effects of non lipolytic ligand function of endothelial Lipase on high density lipoprotein metabolism invivo. J Biol Chem.

[20] Choi SY, Hirata K, Ishida T, Quertermous T, Cooper AD (2002). Endothelial lipase: A new Lipase on the block. J Lipid Res.

